# Strong Antimicrobial Activity of Highly Stable Nanocomposite Containing AgNPs Based on Water-Soluble Triazole-Sulfonate Copolymer

**DOI:** 10.3390/pharmaceutics14010206

**Published:** 2022-01-16

**Authors:** Alexander Pozdnyakov, Artem Emel’yanov, Anastasiya Ivanova, Nadezhda Kuznetsova, Tat’yana Semenova, Yuliya Bolgova, Svetlana Korzhova, Olga Trofimova, Tat’yana Fadeeva, Galina Prozorova

**Affiliations:** 1 A.E. Favorsky Irkutsk Institute of Chemistry, Siberian Branch, Russian Academy of Sciences, 664033 Irkutsk, Russia; emelyanov@irioch.irk.ru (A.E.); ivanova93@irioch.irk.ru (A.I.); nkuznetsova@irioch.irk.ru (N.K.); semjenova@irioch.irk.ru (T.S.); omtrof1@irioch.irk.ru (Y.B.); korzhova@irioch.irk.ru (S.K.); omtrof@irioch.irk.ru (O.T.); prozorova@irioch.irk.ru (G.P.); 2 Irkutsk Scientific Center of Surgery and Traumatology, 664003 Irkutsk, Russia; fadeeva05@yandex.ru

**Keywords:** hydrophilic nanocomposite, antimicrobial activity, silver nanoparticles, 1-vinyl-1,2,4-triazole, vinylsulfonic acid sodium salt

## Abstract

A new hydrophilic polymeric nanocomposite containing AgNPs was synthesized by chemical reduction of metal ions in an aqueous medium in the presence of the copolymer. A new water-soluble copolymer of 1-vinyl-1,2,4-triazole and vinylsulfonic acid sodium salt (poly(VT-co-Na-VSA)) was obtained by free-radical copolymerization and was used as a stabilizing precursor agent. The structural, dimensional, and morphological properties of the nanocomposite were studied by UV–Vis, FTIR, X-ray diffraction, atomic absorption, transmission and scanning electron microscopy, dynamic and electrophoretic light scattering, gel permeation chromatography, thermogravimetric analysis, and differential scanning calorimetry. Hydrodynamic diameter of macroclubs for the copolymer was 171 nm, and for the nanocomposite it was 694 nm. Zeta potential for the copolymer was −63.8 mV, and for the nanocomposite it was −70.4 mV. The nanocomposite had strong antimicrobial activity towards Gram-negative and Gram-positive microorganisms: MIC and MBC values were in the range of 0.25–4.0 and 0.5–8.0 μg/mL, respectively.

## 1. Introduction

Nanocomposites containing silver nanoparticles (AgNPs) exhibit effective antimicrobial activity and cytotoxic properties and are widely used in biomedicine [1,2,3,4,5,6]. Therefore, it is relevant and important to continue intensive studies of the processes of AgNPs formation and their biological activity. The nanoscale state of AgNPs significantly changes their chemical, physical, optical, and biological properties and promotes the development of effective drugs based on them with antimicrobial, antifungal, antiviral, antitumor, antioxidant, and cardioprotective effects [1,2,3,4,7,8,9,10].

AgNPs could be effective nano-radiosensitizers for glioma targeting treatment [11]. A wide range of biological activity of AgNPs contributes to the efficiency of their use to modify orthopedic implants, to prevent infection, and for the manufacture of antibacterial coatings in medical devices and instruments [12,13]. The AgNP nanocomposites with antibacterial activity showed an inhibition effect against biofilm formation and have potential application in food packaging [14,15].

Particular attention is paid to the development of ecofriendly, nontoxic methods of AgNPs synthesis, without the use of high temperature, using different biological sources (plant extracts, yeast, bacteria) [3,7,8,9,13,14,15,16,17,18]. Developing methods for the formation of AgNPs with high stability of the aggregate nanoscale state is an important task of modern medical chemistry. To solve it, various methods for reducing silver ions and stabilizing the obtained particles are intensively studied. Various polymers, both synthetic (polystyrene, polyvinylpyrrolidone, polyethylene glycol, polyacrylamide, polyvinyltriazole, etc.) and natural (cellulose, gelatin, etc.) exhibit high stabilizing activity in the formation of AgNPs [15,19,20,21,22,23,24,25,26,27,28,29,30,31,32].

The presence of macromolecules that actively interact with metal nanoparticles and prevent their aggregation help polymers provide stability. We have previously shown that copolymers of 1-vinyl-1,2,4-triazole, depending on the functional composition, possess valuable properties (hydrophilicity, thermal stability, complexing ability, chemical stability, biocompatibility, non-toxicity, etc.) and are capable of effectively stabilizing nanoparticles of various metals [20,21,33].

In this study, we report on the synthesis of a new water-soluble copolymer based on 1-vinyl-1,2,4-triazole and sodium salt of vinyl sulfonic acid, as well as a silver-containing nanocomposite based on it. The sodium salt of vinyl sulfonic acid is involved in the copolymerization reaction with 1-vinyl-1,2,4-triazole with the goal of increasing the functionality of the new copolymer. Along with triazole fragments, negatively charged sulfonate groups are introduced into macromolecules, which contribute to an increase in the number of coordination centers of metal nanoparticles and a significant increase in the stabilizing ability of the copolymer. At the same time, the spectrum of useful properties of the polymer matrix is expanding, since it is known that blood-compatible drug carriers, which reduce protein absorption and platelet adhesion, as well as drug delivery systems have been developed on the basis of polyvinyl sulfonic acid [34,35,36,37,38,39,40].

Along with the synthesis of a new copolymer of 1-vinyl-1,2,4-triazole with the sodium salt of vinyl sulfonic acid, we discuss the features of the formation of a new hydrophilic silver-containing nanocomposite based on it. We also discuss the results of the study of the main physicochemical, structural, optical, and thermal properties of the copolymer and nanocomposite, as well as their antimicrobial activity against various strains of Gram-negative and Gram-positive microorganisms.

## 2. Materials and Methods

### 2.1. Materials

1-Vinyl-1,2,4-triazole was synthesized and purified according to method [41]. Na-VSA (98.9%, Sigma-Aldrich, Munich, Germany) was used as comonomer without further cleaning. AgNO_3_ (99.9%, Sigma-Aldrich) was used as a precursor to nanoparticles in the generation of AgNPs. NaBH_4_, azobisisobutyronitrile (AIBN), and DMSO (Sigma-Aldrich) were used as the reducing agent, radical initiator, and solvent, respectively.

### 2.2. Poly(VT-co-Na-VSA)

Poly(VT-co-Na-VSA) was synthesized by free-radical copolymerization of 1-vinyl-1,2,4-triazole (4.33 mL, 0.05 mol) and vinylsulfonic acid sodium salt (5.53 mL, 0.05 mol) in DMSO (20 mL) under the action of AIBN (0.16 g, 1.0 mmol) as initiator. Compounds were placed in a glass ampule in an argon atmosphere. The solution was then degassed by three freeze–evacuate–thaw cycles. The ampule was flame-sealed under vacuum and placed in a dry-air thermostat, which was stirred at 70 °C. After 24 h the ampule was immersed in iced water and then opened. The reaction mixture was dissolved in DMSO and extracted with acetone. The copolymer was dried over P_2_O_5_ at 50 °C in vacuo to a constant weight. The product was dissolved in water, purified by dialysis for 48 h through a cellulose membrane with a pore size of 5 kDa (CelluSep H1, MFPI, Seguin, TX, USA), and freeze-dried to yield the copolymer as a white powder (the product yield was 76%). Poly(VT-co-Na-VSA) is soluble in water and dipolar organic solvents (DMF and DMSO). The number average (Mn) and the weight average (Mw) molecular weights of the copolymer were 21,311 and 39,854 Da, respectively. The polydispersity index (Mw/Mn) of the copolymer was 1.87. The obtained hydrophilic poly(VT-co-Na-VSA) was used as a stabilizing agent for AgNPs in the synthesis of a nanocomposite.

### 2.3. AgNPs Nanocomposite

A solution (1 mL) of AgNO_3_ (0.5 mmol) in NH_4_OH (25%) was added to poly(VT-co-Na-VSA) (1.1 g, 10.0 mmol) in water (15 mL). The mixture was intensely stirred for 30 min at 20 °C. NaBH_4_ (0.04 g, 1.0 mmol) in deionized water (5 mL) was added dropwise. The stirring was continued at room temperature for 8 h. The resulting product was extracted with ethanol, then filtered and purified by dialysis against water through a cellulose membrane (5 kDa, CelluSep H1, MFPI). The product was further freeze-dried to yield the nanocomposite as a dark-brown powder (yield was 77%). The AgNPs nanocomposite was soluble in water and dipolar organic solvents (DMSO and DMF). The silver content in the nanocomposite was 4.4%.

### 2.4. Characterization

Elemental analysis was performed on a Flash EA 1112 Series analyzer. Fourier transform infrared (FTIR) spectra were recorded on a Varian 3100 FTIR spectrometer in KBr pellets (Varian, Palo Alto, CA, USA). Ultraviolet–visible spectra were run on a Shimadzu UV-2450 (Shimadzu Corporation, Kyoto, Japan). The silver content in the nanocomposite was determined using the atomic absorption analysis method (AAnalyst 200 instrument, Perkin Elmer Inc., Waltham, MA, USA). SEM microphotographs were obtained using a scanning electron microscope (HITACHI TM 3000, detector SDD XFlash 430-H). TEM microphotographs were obtained on a transmission electron microscope (Leo 906E, Zeiss, Oberkochen, Germany). X-ray diffraction patterns were obtained on a powder diffractometer (D8 Advance, Bruker Corporation, Billerica, MA, USA). The sedimentation stability of the sols was evaluated visually and by ultraviolet–visible spectra. The molecular weight of the copolymer was determined by gel permeation chromatography at 50 °C using a Shimadzu LC-20 Prominence system fitted with a differential refractive index detector Shimadzu RID-20A and column Agilent PolyPore 7.5 × 300 mm (PL1113-6500). N,N-Dimethylformamide solution was used as an eluent with a flow rate of 1 mL/min. Dissolution of samples was performed at 50 °C for 24 h with stirring. Calibration was carried out using a series of standard Polystyrene High EasiVials (PL2010-0201), consisting of 12 samples with molecular weights from 162 to 6,570,000 g/mol. The hydrodynamic particle diameter and zeta potential were determined using a ZetaPALS Zeta Potential Analyzer with a BI-MAS module (Brookhaven Instruments Corporation, Holtsville, NY, USA). The measurements were carried out in deionized water (substance concentration 0.1 mg/mL) in thermostated cuvettes with an operating temperature of 25 °C and an angle of detection of scattered light equal to 90°. Thermogravimetric analysis and differential scanning calorimetry were performed using an STA 449 Jupiter thermal analyzer (Netzsch, Selb, Germany) in an atmosphere of air at a heating rate of 10 °C per min from 25 °C to 1000 °C. The weight of the samples was 5 mg.

### 2.5. Antimicrobial Activity

Minimum inhibitory concentrations (MIC) and minimum bactericidal concentrations (MBC) for poly(VT-co-Na-VSA) and the nanocomposite based on it, containing 4.4% AgNPs, were determined against *Escherichia coli* (ATCC 25,922), *Pseudomonas aeruginosa* (ATCC 27,853), *Klebsiella pneumoniae* (ATCC 700,603), *Staphylococcus aureus* (ATCC 25,923), and *Enterococcus faecalis* (ATCC 29,212) using a serial dissolution method [42]. The initial solutions contained 1000 μg of the sample in 1 mL of water. From these solutions, two-fold concentrations of the drug were prepared in a liquid nutrient medium with a final concentration of the microorganism 5 × 10^5^ CFU/mL (using 8–11 tubes of 1 mL volume). Test organisms were suspended in a concentration of 0.5 McFarland standard (1.5 × 10^8^ CFU/mL) prepared from an agar culture in sterile isotonic sodium chloride solution with densitometer Densimat (Bio Merieux, Marcy-l’Etoile, France). To obtain the required inoculum (5 × 10^5^ CFU/mL), 50 μL of a bacterial suspension containing 10^6^ CFU/mL was added to each tube. The control tube contained 1 mL of broth without preparation and 50 µL of culture for each tested strain. The inoculates were incubated in a normal atmosphere at 35 °C for 18–24 h. The results were evaluated visually, determining the presence or absence of growth in a medium containing a test sample with different concentrations (Table 1 and Table 2).

## 3. Results and Discussion

### 3.1. Copolymerization of VT with Na-VSA

**Figure 1:**
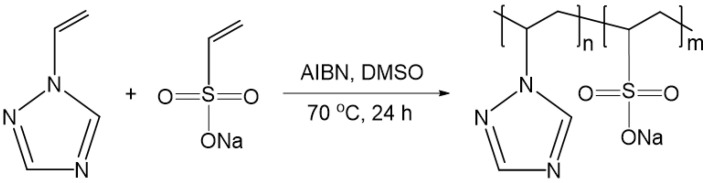
Synthesis of poly(VT-co-Na-VSA).

Copolymerization of 1-vinyl-1,2,4-triazole with vinylsulfonic acid sodium salt was carried out in DMSO solution in the presence of an initiator (AIBN) at 70 °C for 24 h, molar ratio of the starting monomers being 50:50 (Figure 1).

**Figure 2:**
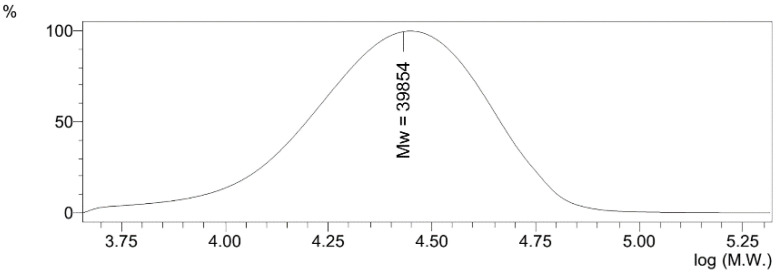
GPC traces of poly(VT-co-Na-VSA).

Structure and composition of the copolymer were established by elemental analysis data, FTIR, ^1^H NMR spectroscopy, SEM microscopy, gel permeation chromatography (GPC), and light scattering measurements. The contents of the VT and Na-VSA fragments in the copolymer were 56 and 44 mol%, respectively. According to the results of gel permeation chromatography, the Mn and Mw of the polymer were 21,311 and 39,854 Da, respectively (Figure 2). The polydispersity index (Mw/Mn) of the copolymer was 1.87.

### 3.2. Synthesis of Nanocomposite

For the synthesis of the nanocomposite, a chemical method was used for the reduction of silver ions from AgNO_3_ in an aqueous medium in the presence of poly(VT-co-Na-VSA) using NaBH_4_ as a reducing agent. AgNO_3_ was previously dissolved in ammonium hydroxide to obtain silver in oxide form. This approach prevented the formation of an insoluble polymer complex, which is formed due to the coordination interaction of silver ions with ligand groups of the copolymer. The reaction mixture was kept for 8 h with constant stirring. The intensive evolution of hydrogen and gradual coloration of the reaction solution from colorless to dark brown was observed. The nanocomposite was purified by dialysis for 48 h, then freeze dried. The resulting polymer nanocomposite was a brown powder, readily soluble in water and dipolar organic solvents (DMF and DMSO). According to atomic absorption spectroscopy, the silver content in the nanocomposite was 4.4%.

### 3.3. FTIR Characterization and X-Ray Diffraction

**Figure 3:**
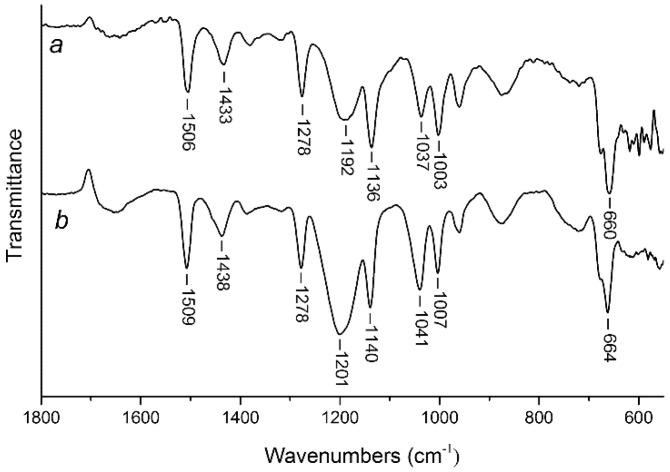
Fourier transform infrared spectra for the starting poly(VT-co-Na-VSA) (**a**) and AgNPs nanocomposite (**b**).

The interaction between the copolymer and AgNPs was studied by FTIR spectroscopy. The spectra of the starting copolymer and nanocomposite with AgNPs are presented in Figure 3.

The stretching and deformation vibrations of the copolymer fragments were observed for the triazole ring at 1506 (C=N), 1433 (C–N), 1278 (N–N), 1136 (C–H), 1003 (C–H), and 660 cm^–1^ (C–N) and for the sulfonate fragment at 1192 (S–O) and 1037 cm^–1^ (S=O). The FTIR spectrum of the nanocomposite was similar to that of the starting copolymer. This indicated the absence of structural transformations in poly(VT-co-Na-VSA) during the synthesis of nanocomposite and indicated that the initial properties of the polymer matrix were preserved. In the FTIR spectrum of the nanocomposite, a shift (by 3–9 cm^−1^) of the absorption bands of the C=N and C–N vibrations of the triazole ring bonds (1509 and 1438, 664 cm^−1^, respectively) and S–O bonds, sulfonate-anion fragments (1201, 1040 cm^−1^) was noted. This shift indicated that the functional groups of both triazole and sulfonate fragments of poly(VT-co-Na-VSA) macromolecules were involved in the coordination of silver ions at the reduction stage and the subsequent stabilization of the resulting AgNPs, preventing further agglomeration of nanoparticles, ensuring their uniform distribution throughout the copolymer matrix and effective stabilization. The structural and dimensional properties of the nanocomposite remained unchanged when it was stored in a dry state and in an aqueous solution in air at room temperature for a long time (>6 months). This indicated a high stabilizing ability of the hydrophilic poly(VT-co-Na-VSA).

**Figure 4:**
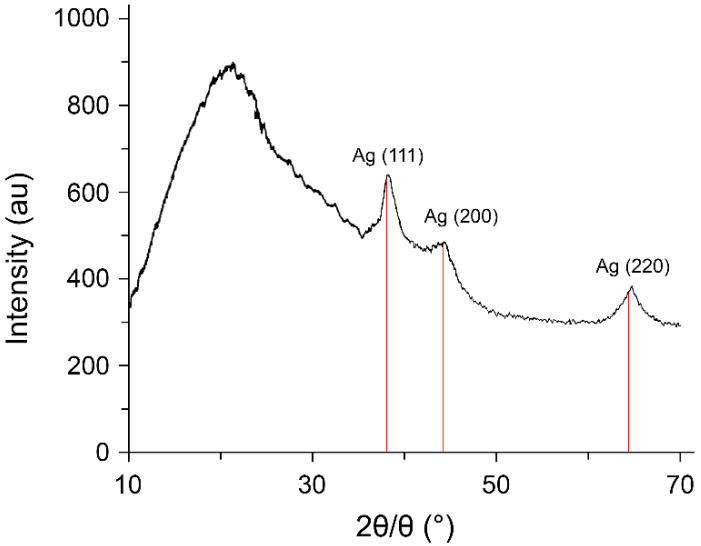
X-ray diffraction pattern for nanocomposite with AgNPs stabilized by poly(VT-co-Na-VSA).

The formation of metallic AgNPs in the nanoscale state in the synthesized nanocomposite was confirmed by the results of X-ray phase analysis. The amorphous halo of the polymer matrix and reflexes of the zerovalent metallic silver were observed in the diffractogram of the nanocomposite (Figure 4).

Data were taken for the 2θ range of 10 to 70° with a step of 0.020° in 1 s at 25 °C. Three main characteristic diffraction peaks at 2θ values of 38.2, 44.3, and 64.5 degrees were identified as metallic silver corresponding to (hkl) values—(111), (200), and (220) planes of the face-centered cubic crystalline structure. The latter was identified by comparison of the values for interplanar spacing and relative intensities with the standard values for metal silver. The X-ray diffraction spectrum of AgNPs was matched with JCPDS card No 04-0783. A diffraction study demonstrated that the average size of the AgNPs (average area of coherent scattering), determined by the Debye–Scherrer method, was 3–12 nm.

### 3.4. UV–Visible Spectroscopy and Electron Microscopy

**Figure 5:**
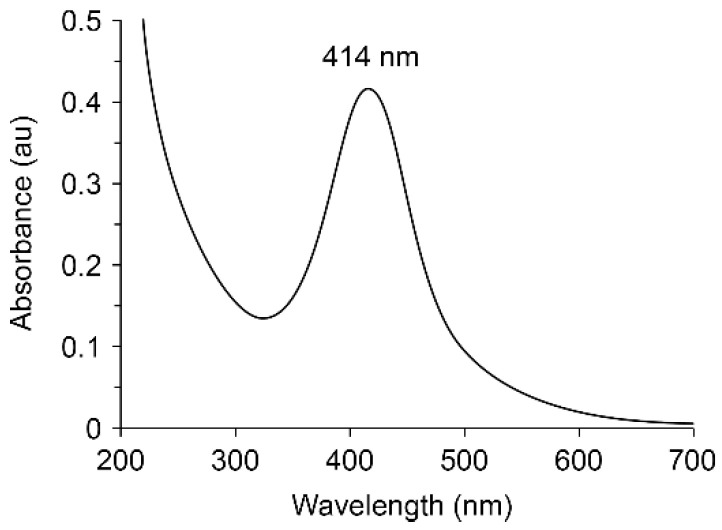
UV–Vis spectra for nanocomposite with AgNPs stabilized by poly(VT-co-Na-VSA).

A band with a maximum at 414 nm appears in the electronic optical absorption spectrum of the nanocomposite, which is characteristic of the plasmon resonance absorption of electrons by metallic AgNPs (Figure 5).

**Figure 6:**
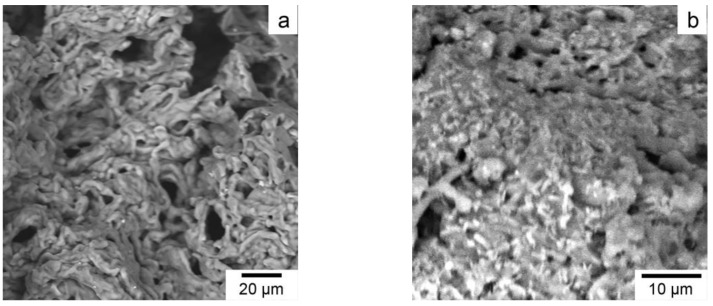
Scanning electron micrograph for the poly(VT-co-Na-VSA) (**a**) and AgNPs nanocomposite (**b**).

The results of scanning electron microscopy indicate different surface morphologies of the copolymer and nanocomposite with AgNPs (Figure 6). The copolymer is characterized by a highly porous surface with numerous channels 4–15 µm in diameter (Figure 6a). The surface morphology of the AgNPs nanocomposite became denser (Figure 6b).

**Figure 7:**
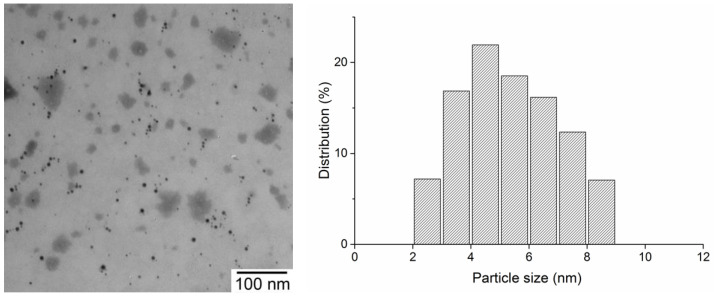
Transmission electron micrograph and respective histogram of AgNPs nanocomposite.

According to transmission electron microscopy data, AgNPs formed in a poly(VT-co-Na-VSA) matrix have a predominantly spherical shape with a size of 2–9 nm and a uniform distribution (Figure 7).

Poly(VT-co-Na-VSA) exhibits high efficiency of AgNPs stabilization, which is due to the high coordination ability of sulfonate and triazole fragments of macromolecules. This is confirmed by the identity of the size of AgNPs and the nature of their distribution in the polymer matrix before and after centrifugation at 10,000 rpm for 20 min.

### 3.5. Dynamic and Electrophoretic Light Scattering

Dynamic light scattering was used to measure the hydrodynamic diameter of macroclubs (*D_h_*) of the initial polymer matrix and nanocomposite in deionized water (substance concentration 0.1 mg/mL).

*D_h_* was measured using a ZetaPALS Zeta Potential Analyzer with a BI-MAS module. The measurements were carried out in thermostated cuvettes with an operating temperature of 25 °C, an angle of detection of scattered light equal to 90°, and a wavelength of 633 nm. The presented results are the average values of three shooting series of 10 cycles. To calculate the hydrodynamic diameter, a standard function was used for spherical particles that do not interact with each other, the size of which is calculated using the Stokes–Einstein formula:

D = k_b_T/6πηR

**Figure 8:**
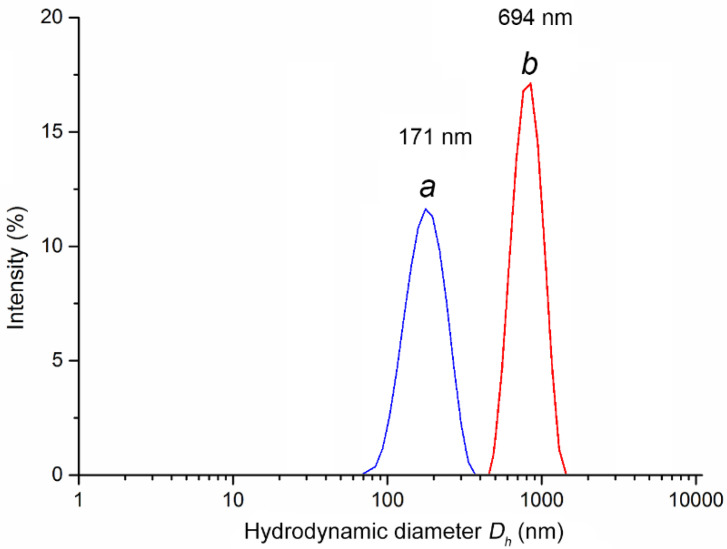
Histogram of the distribution over the hydrodynamic diameter *D_h_* of the scattering particles for the initial copolymer (**a**) and the AgNPs nanocomposite (**b**).

The histograms are characterized by a monomodal size distribution of macroclubs with a maximum corresponding to the effective hydrodynamic diameter of scattering particles of 171 and 694 nm for the copolymer and nanocomposite, respectively (Figure 8).

According to gel permeation chromatography, the number average molecular weights of the copolymer were 21,311 Da. For this molecular weight, the diameter of the scattering particles should not exceed 10 nm. It can be assumed that PVI macromolecules are associated in an aqueous solution. This indicates that large supramolecular structures are formed in water due to the intermolecular interaction of individual macromolecules. The formation of such large associates probably occurs due to the electrostatic interaction of charged groups belonging to different molecular chains of the copolymer. As in the case of the poly-1-vinyl-1,2,4-triazole [43], the triazole groups of the copolymer can partially protonate, providing a positive charge on the macromolecule. Thus, self-assembled structures can be formed due to interchain ionic bonds involving sulfate and triazole units in an ionized state.

Electrophoretic light scattering with phase analysis was used to determine the zeta potential of the initial polymer matrix and the synthesized AgNPs nanocomposite. The zeta potential of the copolymer was −63.8 mV, and the nanocomposite was −70.4 mV. Sulfonate fragments of macromolecules are in an ionized state in an aqueous medium. During the synthesis of a nanocomposite, anionic groups interact with the positively charged surface of silver nanoparticles, which ensures effective stabilization of metal nanoparticles at the early stages of their formation and leads to restriction of their growth by the formation of protective polymer layers.

### 3.6. Thermogravimetric Analysis and Differential Scanning Calorimetry

**Figure 9:**
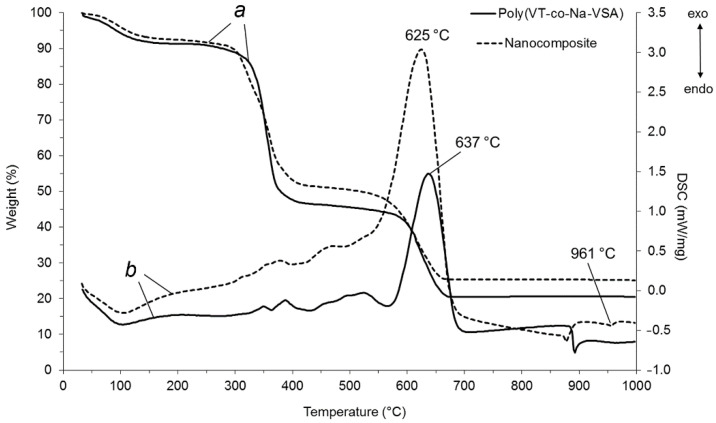
Thermogravimetric (**a**) and differential scanning calorimetry (**b**) curves for the starting poly(VT-co-Na-VSA) and AgNPs nanocomposite.

According to thermogravimetric analysis and differential scanning calorimetry (DSC) data, the resulting nanocomposite is characterized by heat resistance to oxidative degradation up to 305 °C (Figure 9).

In the temperature range 60–160 °C, the mass of the nanocomposite decreased by 7.8% due to the release of adsorbed water, which was accompanied by a corresponding endothermic effect on the DSC curve and the appearance of a signal in the mass spectrum at a mass number of 18. The sample weight loss of 48.5% in the range of 320–425 °C was due to the detachment of the triazole and sulfone fragments from the macromolecular chain and subsequent oxidation to H_2_O, CO_2_, and NO_2_. This was confirmed by the appearance of two exothermic peaks on DSC and signals with the corresponding mass numbers (18, 44, 46) in the mass spectrum. The greatest weight loss of the sample (75%) occurred at 570–710 °C due to combustion of the carbon skeleton with predominant release of CO_2_ (mass number 44). An intense exothermic peak appeared on the DSC curve with a maximum at 625 °C. The endothermic peak at 961 °C corresponds to the melting of metallic silver. It should be noted that the thermal stability of the nanocomposite is 15 °C lower than that of the initial copolymer poly(VT-co-Na-VSA), which is due to the catalytic features of AgNPs, leading to a decrease in the activation energy of thermal destruction and oxidation of the polymer matrix.

### 3.7. Antimicrobial Activity

According to microbiological studies, the AgNPs nanocomposite had high antimicrobial activity against various strains of Gram-negative and Gram-positive bacteria (Table 1). The AgNPs nanocomposite was characterized by MIC and MBC values in the range of 0.25–4.0 and 0.5–8.0 μg/mL, respectively.

**Figure 10:**
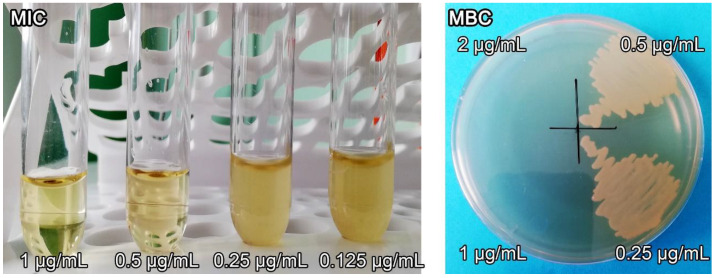
Minimum inhibitory and minimum bactericidal concentrations of AgNPs nanocomposite for *Escherichia coli*.

The nanocomposite was most effective against *P. aeruginosa*, the growth of which was suppressed at a low concentration of 0.25 μg/mL. The MIC and MBC values of the AgNPs nanocomposite in relation to the Gram-negative bacteria *E. coli* (ATCC 25,922) were 0.5 and 1.0 μg/mL, respectively (Figure 10).

The MIC and MBC values of the AgNPs nanocomposite with respect to Gram-positive microorganisms *E. faecalis* (ATCC 29,212) were slightly higher and amounted to 4.0 and 8.0 μg/mL, respectively. It should be noted that the parent poly(VT-co-Na-VSA) did not exhibit antimicrobial effects at all tested concentrations (Table 2).

Consequently, the antimicrobial activity of the nanocomposite is due to the presence of nanosized narrowly dispersed AgNPs in the copolymer matrix. The results indicate a higher antimicrobial activity of the AgNPs nanocomposite in the poly(VT-co-Na-VSA) matrix compared to silver-containing nanocomposites based on the homopolymer of 1-vinyl-1,2,4-triazole [20] and poly(VT-co-VP) [21] synthesized by us earlier.

## 4. Conclusions

We synthesized a new water-soluble silver-containing nanocomposite based on a copolymer of 1-vinyl-1,2,4-triazole and a sodium salt of vinyl sulfonic acid. The resulting AgNPs are characterized by narrowly dispersed sizes (2–9 nm) and uniform distribution in the copolymer matrix. The copolymer and AgNPs nanocomposite are characterized by a monomodal size distribution of macroclubs with a maximum corresponding to the effective hydrodynamic diameter of the scattering particles at 171 and 694 nm, respectively, established by dynamic light scattering. The zeta potential of the copolymer (−63.8 mV) and the nanocomposite with AgNPs (−70.4 mV) was determined. The nanocomposite with AgNPs is highly stable to thermal oxidative degradation (up to 305 °C). The AgNPs nanocomposite exhibits high antimicrobial activity against various strains of Gram-negative and Gram-positive microorganisms and is characterized by low values of the minimum inhibitory and bactericidal concentrations, 0.25–4.0 and 0.5–8.0 μg/mL, respectively. The advantage of the nanocomposite is also its high stability when stored in dry form and in aqueous solution for 6 months. The synthesized water-soluble AgNPs nanocomposite based on the poly(VT-co-Na-VSA) copolymer is promising for the development of effective antiseptic and antimicrobial materials used for the treatment of a number of infectious diseases.

## Figures and Tables

**Table 1:** Antimicrobial activity of AgNP nanocomposite.

Microorganisms	MIC/MBC, μg/mL								
	500-32	16	8	4	2	1	0.5	0.25	0.125
*Escherichia coli* ATCC 25,922	− −/− −	− −/− −	− −/− −	− −/− −	− −/− −	− −/− −	− − /+ +	+ +/+ +	+ +/+ +
*Pseudomonas aeruginosa* ATCC 27,853	− −/− −	− −/− −	− −/− −	− −/− −	− −/− −	− −/− −	− −/− −	− − /+ +	+ +/+ +
*Klebsiella pneumoniae* ATCC 700,603 (EBSL)	− −/− −	− −/− −	− −/− −	− − /+ +	+ +/+ +	+ +/+ +	+ +/+ +	+ +/+ +	+ +/+ +
*Staphylococcus aureus* ATCC 25,923	− −/− −	− −/− −	− −/− −	− −/− −	− − /+ +	+ +/+ +	+ +/+ +	+ +/+ +	+ +/+ +
*Enterococcus faecalis* ATCC 29,212	− −/− −	− −/− −	− −/− −	− − /+ +	+ +/+ +	+ +/+ +	+ +/+ +	+ +/+ +	+ +/+ +

Note: (−) Absence of test-strains growth; (+) presence of growth of test strains.

**Table 2:** Antimicrobial activity of poly(VT-co-Na-VSA).

Microorganisms	MIC/MBC, μg/mL								
	500-32	16	8	4	2	1	0.5	0.25	0.125
*Escherichia coli* ATCC 25,922	+ +/+ +	+ +/+ +	+ +/+ +	+ +/+ +	+ +/+ +	+ +/+ +	+ +/+ +	+ +/+ +	+ +/+ +
*Pseudomonas aeruginosa* ATCC 27,853	+ +/+ +	+ +/+ +	+ +/+ +	+ +/+ +	+ +/+ +	+ +/+ +	+ +/+ +	+ +/+ +	+ +/+ +
*Klebsiella pneumoniae* ATCC 700,603 (EBSL)	+ +/+ +	+ +/+ +	+ +/+ +	+ +/+ +	+ +/+ +	+ +/+ +	+ +/+ +	+ +/+ +	+ +/+ +
*Staphylococcus aureus* ATCC 25,923	+ +/+ +	+ +/+ +	+ +/+ +	+ +/+ +	+ +/+ +	+ +/+ +	+ +/+ +	+ +/+ +	+ +/+ +
*Enterococcus faecalis* ATCC 29,212	+ +/+ +	+ +/+ +	+ +/+ +	+ +/+ +	+ +/+ +	+ +/+ +	+ +/+ +	+ +/+ +	+ +/+ +

Note: (+) presence of growth of test strains.

## Data Availability

On request.
